# Cautious Optimism Warranted for Stem Cell-Derived Islet Transplantation in Type 2 Diabetes

**DOI:** 10.3389/ti.2024.13358

**Published:** 2024-07-26

**Authors:** Hanne Scholz, Valeria Sordi, Lorenzo Piemonti

**Affiliations:** ^1^ Department of Transplant Medicine and Institute for Surgical Research, Oslo University Hospital, Oslo, Norway; ^2^ Hybrid Technology Hub Centre of Excellence, Institute of Basic Medical Science, University of Oslo, Oslo, Norway; ^3^ Diabetes Research Institute, IRCCS San Raffaele Hospital, Milan, Italy; ^4^ Clinic Unit of Regenerative Medicine and Organ Transplants, IRCCS Ospedale San Raffaele, Milan, Italy

**Keywords:** type 2 diabetes, islet cells, induced pluripotent stem cells, islet transplantation, autologous

A 59 years old man in China has become the first patient to receive functional autologous islet tissue differentiated from inducible pluripotent stem cells (iPSC) [1]. In a previous case, injecting a poorly differentiated autologous iPSC product into the deltoid muscle led to a malignant teratoma [2] hampering proper evaluation due to insufficient information.

Wu et al reported on their proof-of-concept and safety study on 30th April 2024, in Cell Discovery [1]. The authors explore the use of endoderm stem cell lines [3] established from the patient’s own peripheral blood mononuclear cells to generate islet tissues (E-islets) *in vitro* to treat a patient diagnosed with type 2 diabetes (T2D) 25 years ago. The patient had previously received a kidney transplant due to end-stage diabetic nephropathy, which required systemic immunosuppressive drugs to prevent rejection. A previously published protocol was used for *in vitro* differentiation of iPSCs into fully functional insulin producing islet cells [3]. A clinical dose defined as 1.2 × 10^6^ IEQs was transplanted intraportally according to the established allogeneic islet transplant procedure [4] with a follow up of glycemic targets, reduction of exogenous insulin, and levels of fasting and meal-stimulated circulating C-peptide/insulin post transplantation. The present study suggests that stem cell-derived islet tissues could effectively restore islet function in a late-stage T2D patient. Additionally, the graft was well-tolerated with no instances of tumor formation or severe adverse events linked to the transplantation.

The novelty of this work lies in four key aspects: 1) the choice of iPSC to generate islets, as opposed to embryonic stem cells used in previously published clinical trials; 2) the utilization of an intermediate clinical grade cell line of endoderm stem cells (EnSCs) to facilitate non-tumorigenic, consistent, large-scale manufacture of the E-islets; 3) the use of autologous, patient-derived stem cells, and 4) the transplantation of a patient with type T2D. The use of human iPSCs as starting material is rapidly emerging and iPSCs have recently become a trusted autologous cell source that could be implemented for the treatment of Parkinson’s disease [5], macular degeneration [6], and MSC-based therapy for steroid-resistant acute graft *versus* host disease [7]. We applaud the team from Shanghai Hospital, China, to conduct and report on this groundbreaking study but emphasize that there are several weaknesses that should be addressed.

To manufacture large-scale clinical-grade cell products of E-islet, multiple quality control (QC) release criteria must be met and reported throughout the process. This begins with generating of a GMP iPSCs clone, continuing with the development of a master cell bank of pancreatic endoderm stem cells (EnSCs), frozen as an intermediate product, and the final release of the drug product (E-islets) for transplantation. The references for the selected specific methods, justification of the timing, the batch size, and the rationale for the quality control platform are not clearly detailed by the authors [8].

Although, the efficiency of *in vitro* differentiation is good, it is not clear if multiple rounds of differentiation are needed to obtain enough yield of E-islets for a clinical batch. The single-cell transcriptomic (SCT) analysis data on E-islets highlights a significant presence of glucagon-positive cells (almost half of the sequenced cells), which is not concordant with the characterization of E-islets by flow cytometry.

A real clinically relevant potency assay is one of the most challenging aspect to achieve for the new wave of biological drugs, also known as advanced therapy medicinal products (ATMPs) [9]. The authors selected a static stimulated insulin secretion assays that is a robust assay for primary human islets, but without any known sensitivity to distinguished quality control for stem cell derived islet like cells [10]. *In vivo* diabetic transplantation model was selected to determine the functionality and immunogenicity of the cells prior to transplantation. Although the authors created both a humanized mouse model using patient-specific blood mononuclear cells and non-human primates to study reversal of diabetes by E-islets, it is difficult to draw definitive conclusions about the function of these islets given the low number of mice used, pre-transplant blood sugar levels <400 mg/dL [11], lack of complete characterization of explanted grafts and short follow-up [12].

The advantage of using autologous iPSCs generated from a patient’s somatic cell is to obtain patient-specific fully major histocompatibility complex (MHC) match. However, derivatives of autologous iPSCs have been shown to be rejected due to neoantigens that arise during *in vitro* cell manipulations and expansion, thereby questioning their immune-evasive potential [13]. The present study uses an autologous approach but since the recipient was already on immunosuppression due to the kidney transplant, this completely masks the immunological response to the autologous tissue, making this setting a wasted opportunity to answer an important question in the field.

Recent advances in differentiation protocols carefully developed over the past decades, represent one of the greatest achievements in the field. However most published protocols have been evaluated by a mixture of pluripotent stem cells generated from embryonic or reprogramming somatic cells into iPSC which makes it difficult to judge the impact on the differentiation. The field is still too young to declare the superiority of one strategy over the other [14].

Using the human islet equivalent number (IEQ) calculation for the dose selection of the stem-cell-derived islet cells is not accurate. The authors use the primary human islets volume-based method to estimate the number of E-islets but human islets are spherical structures ranging in size from 91 to 290 μm in diameter [15]. In contrast, the E-islets exhibit a uniform morphology consisting of smaller islet cells (one hundred µm), which impacts the tissue volume. Therefore, we advocate for reporting the clinical doses based on single cell counts using validated methods before allowing for 3D generation prior to transplantation.

One of the strengths of the paper is the thorough and comprehensive analysis of the safety of stem-derived cells, and it is hoped that this will serve as a reference for future studies.

From the clinical point of view, the outcome of the endoderm stem cell-derived islet tissue transplantation is challenging to fully assess due to several key factors.

First, the definition of T2D in this context raises several questions. The early onset of the disease at 24 years of age is atypical for classic type 2 diabetes, which is more commonly diagnosed in older adults. Furthermore, the absence of precise information about the patient’s previous medical history, including details such as family history, weight, insulin resistance, C-peptide levels, and autoimmunity at the time of disease onset, makes the classification of this case as T2D labile. This is especially concerning when considering the metabolic features of the patient in comparison to other cohorts of individuals with T2D (Figure 1) [16–18]. This diagnostic uncertainty is a common challenge encountered also in the context of pancreas transplantation for presumed T2D [19]. In such cases, the absence of comprehensive baseline data can pose challenges in accurately distinguishing T2D from other forms of the disease, including genetic subtypes like MODY or LADA.

**FIGURE 1:**
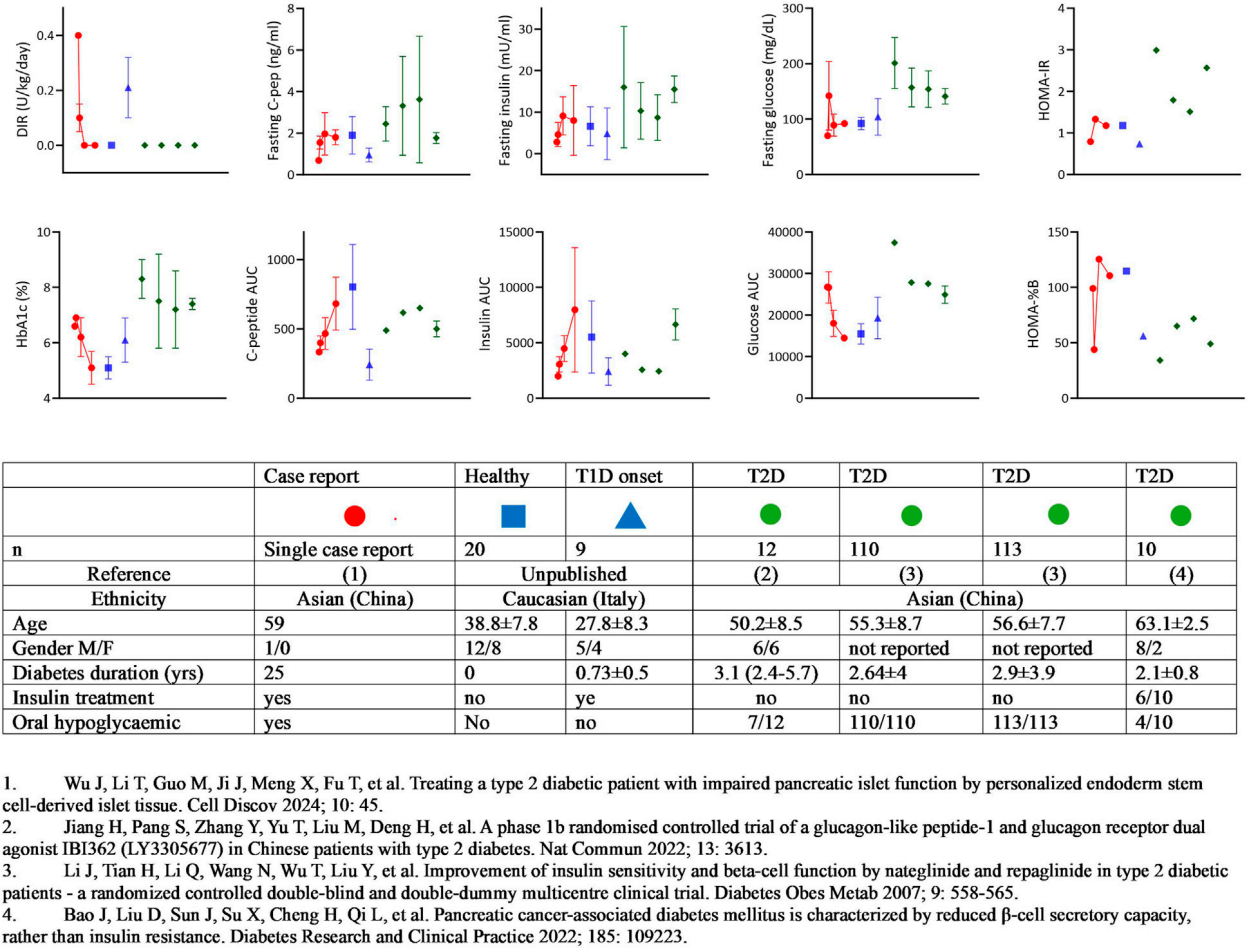
Summary of metabolic features: stem-cell-derived islet cells transplantation vs T1D and T2D Cohorts. The figure summarizes the metabolic characteristics of the patient who received autologous islet tissue differentiated from induced pluripotent stem cells, in comparison with cohorts of individuals with type 1 diabetes (T1D) and type 2 diabetes (T2D). The data points for the case report patient are represented by the red circle, with four descriptive time points provided: 1) baseline; 2) mean value between baseline and 12 weeks (still on insulin treatment); 3) mean value between 12 and 52 weeks (still on antidiabetic treatment; 4) mean value after discontinuation of any diabetic treatment. For reference, data from healthy adults or T1D patients within the first year of onset followed at Ospedale San Raffaele in Milan are shown in blue symbols, and four cohorts of Chinese T2D patients reported in the literature are represented by green symbols. The table accompanying the figure provides the characteristics of the different cohorts.

Secondly, despite the patient being described as having “poor glycemic control,” the reported data does not support this characterization and raises some ethical concerns. In fact, all the metabolic parameters of glucose control at baseline appear to be well within the target range, considering the current clinical goals [20]. This discrepancy raises doubts about the risk-benefit balance of the stem cell-derived islet tissue transplantation approach. Given the patient’s stable glycemic control, alternative strategies could potentially achieve similar or even better outcomes without the risks and complications associated with a “first in man” experimental procedure. Such alternative approaches could include optimizing insulin dose titration or exploring the use of SGLT-2 inhibitors or GLP-1 agonists, which have demonstrated efficacy in the management of type 2 diabetes. Moreover, islet allotransplantation is a well-established procedure for patients having a kidney transplantation [21] and has been translated from an experimental procedure into a validated therapy during 25 years of research contribution [22]. In this context, pending the real need, the islet after kidney transplantation strategy should have been the first option considered as a potentially less risky alternative for this patient.

It is also difficult to distinguish the contribution of the transplanted tissue to the clinical outcome during the follow-up period. Residual function before the infusion was quite substantial with a 2 nmol/L C-peptide. Overall, there is a discrepancy in the early results with insulin independence achieved by 12 weeks, despite glucose levels remaining as high as before the transplant, and C-peptide levels at 12 weeks showing minimal difference compared to before the transplant. Significant clinical impact on glucose variability is also reported within 12 weeks, and even within 2 weeks after tissue infusion. However, this immediate improvement is not accompanied by a corresponding significant increase in C-peptide AUC or a decrease in glycated hemoglobin. This suggests that other factors may have influenced the outcomes, such as trial effects, changes in timing and carbohydrate intake [23] (as indicated by early-phase gastrointestinal disturbances and a 5 kg weight loss), or simply the reduction or suspension of insulin treatment. More congruent appears the improvement of insulin secretion and the improved glucose control in the following weeks, even if it is not possible to distinguish the endogenous contribution and any other potential confounding factors, such as the reported tapered drug administration of tacrolimus.

In conclusion, the field of stem-cell research are making substantial scientific advancements in developing a new generation of iPSCs as an unlimited source for generating cell types such as pancreatic beta cells and/or islet cells. Maintaining optimism is encouraged. However, it remains unclear from the present study whether islet tissue generated from autologous stem cells is an efficient beta cell replacement therapy and if the immunogenic profile of autologous EnSCs used to generate E-islets triggers immune responses. Nevertheless, we believe that cell therapy has the potential to provide a markedly superior alternative to insulin therapy for patients with T1D. More data will be needed before expanded indications for T2D can be established.

## Data Availability

The original contributions presented in the study are included in the article/Supplementary Material, further inquiries can be directed to the corresponding author.
